# Peri-partum respiratory management of pregnant women with neuro-muscular disorders: a prospective observational study (IT-NEUMA-Pregn study)

**DOI:** 10.1186/s12871-023-02307-6

**Published:** 2023-10-13

**Authors:** Fabrizio Racca, Yaroslava Longhitano, Christian Zanza, Mario Giosuè Balzanelli, Gaetano Draisci, Paolo Augusto Stoia, Evelina Gollo, Mariella Maio, Claudia Grattarola, Marinella Astuto, Antonello Ciccarelli, Giulia Racca, Tatsiana Romenskaya, Benedetta Giordano, Alessandra Serraino, Valeria Ada Maria Sansone, Cesare Gregoretti, Giorgio Conti, Fabio Piccolella, Rosanna Vaschetto

**Affiliations:** 1 Department of Anesthesiology and Intensive Care, Azienda Ospedaliera SS. Antonio E Biagio E Cesare Arrigo, Alessandria, Italy; 2 Division of Anesthesia and Critical Care Medicine, Azienda Ospedaliera Ordine Mauriziano, Turin, Italy; 3 Department of Anesthesiology and Perioperative Medicine, University of Pittsburgh, Pittsburgh, PA USA; 4 Post Graduate School of Geriatric Medicine, University of Rome “Tor Vergata”, Rome, Italy; 5 Institute of Anesthesia and Intensive Care, Fondazione Policlinico Universitario Agostino Gemelli IRCCS, Università Cattolica del Sacro Cuore, Rome, Italy; 6 Anesthesiology and Intensive Care Unit, ASST Grande Ospedale Metropolitano Niguarda Ca’Granda, Milan, Italy; 7 Department of Anesthesiology and Intensive Care A.O.U. Città Della Salute E Della Scienza Di Torino, Turin, Italy; 8 Anesthesiology and Intensive Care Unit, Istituto Giannina Gaslini, Genova, Italy; 9 Dipartimento Chirurgia Generale e Specialità Medico Chirurgiche, A.O.Universitaria “Policlinico-Vittorio Emanuele”, Università Degli Studi Di Catania, Catania, Italy; 10 Department of Movement, Human, and Health Sciences - Division of Health Sciences, University of Rome “Foro Italico, Rome, Italy; 11 Department of Translational Medicine, University of Eastern Piedmont, Novara, Italy; 12 Department of Human Neurosciences, Sapienza University of Rome, Rome, Italy; 13 The NEMO Clinical Center in Milan, Neurorehabilitation Unit, University of Milan– ERN for Neuromuscular Diseases, Milan, Italy; 14 Department of Surgical, Oncological and Oral Science (Di.Chir.On.S.), University of Palermo, Palermo, Italy; 15 Fondazione Istituto “G. Giglio” Cefalù, Palermo, Italy

**Keywords:** Pregnancy, Neuromuscular diseases, Postoperative respiratory complications, Non-invasive ventilation, Mechanical cough device

## Abstract

**Background:**

Pregnant women with neuromuscular diseases (NMDs) often display respiratory muscle impairment which increases the risk for pulmonary complications (PCs). The aim of this study was to identify pregnant NMDs patients with pulmonary risk factors and to apply in these women non-invasive ventilation (NIV) combined with mechanical insufflation-exsufflation (MI-E) in the peri-partum period.

**Methods:**

We conducted a multicenter observational study on women with NMDs undergoing cesarean section or spontaneous labor in a network of 7 national hospitals. In these subjects we applied a protocol for screening and preventing PCs, and we evaluated PCs rate, maternal and neonatal outcome.

**Results:**

Twenty-four patients out of the 94 enrolled pregnant women were at risk for PCs and were trained or retrained to use NIV and/or MI-E before delivery. After delivery, 17 patients required NIV with or without MI-E. Despite nine out of the 24 women at pulmonary risk developed postpartum PCs, none of them needed reintubation nor tracheostomy. In addition, the average birth weight and Apgar score were normal. Only one patient without pulmonary risk factors developed postpartum PCs.

**Conclusion:**

This study showed the feasibility of applying a protocol for screening and treating pregnant NMDs women with pulmonary risk. Despite a PCs rate of 37% was observed in these patients, maternal and neonatal outcome were favorable.

**Supplementary Information:**

The online version contains supplementary material available at 10.1186/s12871-023-02307-6.

## Introduction

Recent improvements in the management of neuromuscular diseases (NMDs) have ameliorated both patient’s quality of life and survival [1–3]. Consequently, both inadvertent and planned pregnancies rate are increased [4].

Pregnancy is associated with physiologic respiratory changes [5–7], which can be poorly tolerated in case of respiratory disease. In particular, the growing fetus impairs diaphragm excursion and rises respiratory muscles load, increasing the risk of respiratory failure. NMDs are a heterogeneous group of diseases and different forms may differ in terms of disease onset, progression and severity [3]. When muscle weakness involves respiratory and bulbar muscles, NMD may lead to hypoventilation and ineffective cough [3]. As a consequence, this subgroup of women may develop pulmonary complications (PCs) during pregnancy [4, 8–12]. Furthermore, abdominal and truncal muscle weakness, which may be present even in mild forms of NMDs, may require a cesarean section, which further increase the risk of peri-partum complications [8, 12, 13].

Recommendations for pregnancy [4] and anesthesia management [14–16] of NMDs patients have been recently issued. In particular, in the multidisciplinary evaluation of these women before delivery, pulmonary assessment is strongly recommended to estimate the risk of PCs and the need for specific management, including non-invasive ventilation (NIV) combined with mechanical insufflation-exsufflation (MI-E) [4]. These treatments can successfully improve hypoventilation and airway secretion clearance averting PCs, prolonged intubation, and tracheostomy [14–18].

To the best of our knowledge, very few cases reported the use of NIV in the peripartum period [9, 19–24]. and the application of MI-E in pregnant women has never been reported.

The primary aim of this study was to evaluate the feasibility of a protocol for identifying pregnant NMDs women with pulmonary risk factors and preventing PCs by applying NIV combined with MI-E in the peri-partum period. The secondary aims were to evaluate the prevalence of pregnant women with respiratory risk factors, the percentage of PCs and the maternal and neonatal outcome. In addition, we assessed the safety and tolerability of using MI-E in pregnant women.

The protocol in brief and the preliminary results of this study have already been published as a commentary [25].

## Methods

### Patients and data collection

This multicenter observational pilot study was approved by our Institutional Review Board (IRB) of AON SS Antonio and Biagio and Cesare Arrigo with code n.175246/AR, and written informed consent was obtained from all subjects. Data were collected from December 2015 to December 2022 in a network of seven national hospitals (IT-NEUMA-Pregn study) and uploaded on a password-protected web database. Consecutive pregnant women with NMDs undergoing cesarean section or spontaneous labor were included into the study. Patients with tracheostomy were excluded.

NMDs is a group of disorders whose site of injury can be at the level of motor neurons peripheral nerve, neuromuscular junction, or skeletal muscle. Still undiagnosed NMD was defined as neuromuscular disorder of unknown etiology. The diagnostic workup was verified by the referring neurologists. Where applicable the genetic diagnosis was recorded.

Pulmonary complications comprehended any of the following findings resulting in the first 7 days after delivery: i) secretion retention (i.e., airway secretion encumbrance that is clinically significant characterized by dyspnea or drop in pulse oximetry which improves with secretions removal), ii) pneumonia, iii) atelectasis, iv) pneumothorax, v) acute respiratory failure, vi) bronchospasm, vii) pleural effusion. They also included: i) invasive mechanical ventilation > 48 h after surgery, ii) re-intubation, iii) need for a tracheostomy.

### Protocol

All patients were treated according to a standardized, shared protocol, which was mainly drawn from the Italian recommendations for anesthesia and perioperative management of patients with NMDs published in 2013 [15]. The study protocol (protocol No. 473, November 4th, 2015) was shared with all centers and received ethical approval at all sites. A physician from each participating center was responsible for data collection; the protocol was explained and demonstrated during two specific educational meetings.

**Fig. 1:**
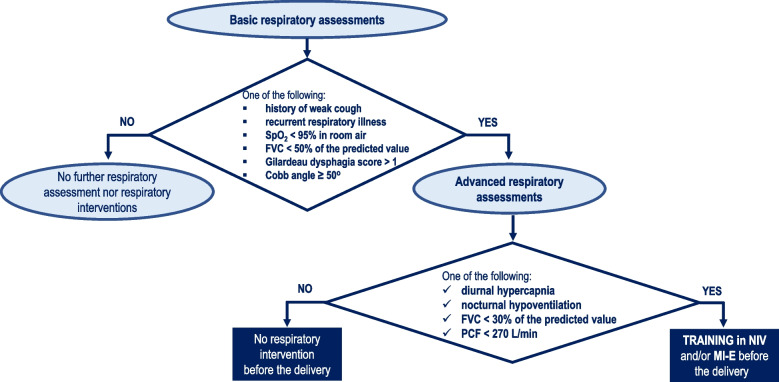
Respiratory management pathway in NMDs pregnant women without respiratory devices dependency. Legend: NMDs, Neuromuscular disorders; FVC, forced vital capacity; SpO2, hemoglobin saturation; PCF, peak cough flow; ABG, arterial blood gas; NIV, non-invasive ventilation; MI-E, mechanical insuflator-exsufflator

Patients were approached at the 28th-30th week of pregnancy. Our protocol included. a respiratory assessment to evaluate the effectiveness of coughing, gas exchange and lung volumes (Fig. 1). Pre-existing respiratory device dependency was also taken into account.

In addition, swallowing and scoliosis were respectively evaluated by Gilardeau dysphagia score [26] and Cobb angle. Patients were classified as at risk for PCs if one of the following conditions was found: *i)* oxygen saturation at room air (SpO_2_) < 95%; *ii)* diurnal or nocturnal hypercapnia; *iii)* central or obstructive apneas; *iv)* history of weak cough or recurrent respiratory illness; *v*) long-term NIV; *vi*) use of cough assistance techniques at home; *vii)* forced vital capacity (FVC) < 50% of predicted value; *viii)* peak cough flow (PCF) < 270 L/min; ix) Gilardeau dysphagia score > 1; *x)* Cobb angle ≥ 50°.

In addition, all patients underwent neurological assessment to confirm the diagnosis, when feasible, and to identify the level of disease progression in each patient. Drug therapy was optimized, in particular for patients with Myasthenia Gravis. Moreover, patients with myopathies underwent careful assessment of heart function including an electrocardiogram and echocardiogram, if not performed in the previous 12 months. Arrhythmias were investigated with an Holter EKG monitoring. Cardiac therapies were also optimized before delivery.

Patients identified at risk for PCs were trained or re-trained to use NIV and/or MI-E before the delivery. Management pathway for women at risk of PCs included a 2-stage assessment of respiratory status (Fig. 1). Trained or re-trained to use NIV and/or MI-E were performed only in case of diurnal or nocturnal hypercapnia, FVC < 30% of predicted value or PCF < 270 L/min..Mechanical insufflation-exsufflation (MI-E) is a technique used to optimize airway clearance in patients who have an impaired cough reflex due to respiratory muscle weakness [27]. This technique improves airway secretions mobilization through the application of rapidly alternating positive and negative pressure, which increases inspiratory and expiratory flows [28]. The respiratory training was performed before delivery in outpatient setting (Table 1). All procedures and training protocols were fully covered by the National Health System.

**Table 1:** NIV and MI-E training performed in outpatient setting before delivery

NIV training	MI–E training
✓ After a thorough explanation of the principles of NIV to the women, NIV was started during short, repetitive periods during the day ✓ The patient was trained by a pulmonologist or an intensivist experienced in the use of NIV ✓ The interface was either a nasal mask or a facial mask ✓ The pressure setting was progressively increased, taking into account the comfort of the woman, to achieve a minimal tidal volume of 8 ml/kg with a good tolerance during at least 30 min period	✓ After a detailed description of the principles of MI-E, the patient was trained by a physiotherapist to the MI–E using a cough assist device where pressures are generated by a two-stage centrifugal blower ✓ Initial inspiratory and expiratory pressures of MI-E were set at a low level (+ 15/-15 cmH2O, respectively). Subsequently, the positive and negative pressures were progressively increased up to a maximum of 40 cmH2O ✓ During insufflation–exsufflation applications face mask, which was firmly applied on the patient’s face, was used

NIV and MI–E training was carried out during a 1-day hospitalization in an outpatient setting, between 1 and 4 weeks prior to the delivery. At the end of the training trial, the patient was discharged home and was instructed to use the NIV with a minimum of 30 min per day, associated with at least one daily MI–E session

*NIV* non-invasive ventilation, *MI-E* mechanical insuflator-exsufflator

In case of intubation for general anesthesia during cesarean section, NIV and MI-E were performed immediately after extubation in patients who already used these devices before pregnancy and in women with preoperative FVC < 30% of predicted and/or PCF < 270 L/min. Patients at risk for PCs, who underwent spinal anesthesia, used NIV and MI-E during cesarean section and in postoperative period when required. Postpartum admission to ICU or HDU was considered in any patient at risk for respiratory or cardiac complications.

The method of delivery and the choice of epidural analgesia for labor, anesthetic technique and postoperative pain management in case of cesarean section were left to the discretion of the multidisciplinary team in charge of the patient. In particular, the mode of delivery was decided with an interdisciplinary approach, evaluating scoliosis severity, respiratory impairment and muscular strength. The choice of epidural analgesia for labor during spontaneous delivery were left to the discretion of the multidisciplinary team in charge of the patient. Regarding the anesthetic management in case of cesarean section, all patients were treated according the Italian recommendations for anesthesia in NMDs [15]. According to these recommendations general anesthesia (GA) was avoided preferring regional anesthesia whenever possible. If GA was unavoidable, ultra-short acting drugs, such as propofol and remifentanil were used. Succinylcholine was never used in all patients with NMDs. The combination of rocuronium and sugammadex replaced succinylcholine if rapid sequence induction was indicated. Furthermore, the administration of halogenated agents in myopathic patients was never used except for those with mitochondrial myopathies.

The influence of pregnancy on the course of disease (e.g., worsening of symptoms during or after the pregnancy) in the months-years following delivery was not assessed because beyond the scope of the present study.

### Statistical analysis

Continuous data were presented as mean and standard deviation or median and interquartile ranges.

The outcomes of the study comprehend the evaluation of the percentages of respiratory peripartum complications, other peripartum complications and the percentage of pregnant women with respiratory risk factors which were expressed as percentage and CI. The hospital and ICU length of stay (LOS) were expressed as average days value and standard variation.

## Results

This study found that among a population of pregnant women a total of 103 consecutive NMDs pregnant women were eligible to enter the study. Nine patients were excluded for incompleteness of data; seven of them did not perform respiratory assessment and post-delivery data were not reported in other two women. Thus, the final number of analyzed patients was 94. Consort flow diagram is shown in Table S3, available in online supplementary material.

### Demographic and clinical characteristics

Myopathies were present in 53% of patients and they were the most frequently represented diseases. Neuromuscular disease diagnosis is shown in detail in Table 2. Demographic and clinical data before delivery are described in Table 3. Results of assessment and management before delivery are shown in Table 4.

**Table 2:** Neuromuscular disease diagnosis

**Diagnosis**	**Patients** (94 cases)
**MOTONEURON DISEASES**	**12**
Spinal Muscular Atrophy	10
Type 2	5
Type 3	5
Amyotrophic Lateral Sclerosis	1
Other Motoneuron Diseases	1
**PERIPHERAL NEUROPATHIES**	**8**
Hereditary Sensory and Motor Neuropathy	2
Chronic Inflammatory Demyelinating Polyneuropathy	4
Other Peripheral Neuropathies	2
**DISORDERS OF NEUROMUSCULAR JUNCTION**	**20**
Myasthenia Gravis	20
**MYOPATHIES**	**50**
**Progressive Muscular Dystrophy**	
Limb-Girdle Muscular Dystrophy	6
Facio-Scapulo-Humeral Muscular Dystrophy	*8*
Myotonic Dystrophy	5
Type 1	3
Type 2	2
*Myotonia Congenita*	3
Central Core Myopathy	1
Metabolic Myopathy	
Mitochondrial Encephalomyopathy	2
Glycogen Storage Disease	2
Others Metabolic Myopathies	1
**Unspecified Myopathies**	22
**STILL UNDIAGNOSED NEUROMUSCULAR DISEASE**	**4**

**Table 3:** Demographic and clinical data before delivery

	**Patients** (94 cases)
**Age** (years)	**33.3 ± 5,1**
**Weight** (Kg)	**71,9 ± 11.0**
**Height (**cm**)**	**163.4 ± 6.7**
**Pre-existing respiratory device dependence** (N. of patients)	**11 (11,7%)**
*NIV*	*10 (10,6%)*
*MI-E*	*8 (8,5%)*
**Scoliosis** (N. of patients)	**17 (18,1%)**
Cobb Angle* 10°- 50°*	*12*
*Cobb Angle 51–90°*	*4*
*Cobb Angle* > *90°*	*1*
***Dysphagia*** (N. of patients)	***5 (5,3%)***
Gilardeau dysphagia *score of 1*	*1*
Gilardeau *dysphagia score of 2*	*3*
Gilardeau *dysphagia score of 3*	*1*
Gilardeau dysphagia *score of 4*	*0*
**GERD** (N. of patients)	***28 (29,8%)***
**Gastrointestinal dysmotility** (N. of patients)	**1 (1,1%)**
**Gestational diabetes** (N. of patients)	**9 (9,6%)**
**Pre-eclampsia** (N. of patients)	**3 (3,2%)**
**Gestational hyperthension** (N. of patients)	**7 (7,4%)**
**Oligohydramnios** (N. of patients)	**1 (1,1%)**
**Gestational hepatosis** (N. of patients)	**1 (1,1%)**

Gilardeau dysphagia score: 0 = able to eat normal diet / no dysphagia; 1 = able to swallow some solid foods; 2 = able to swallow only semi solid foods; 3 = able to swallow liquids only; 4 = unable to swallow anything / total dysphagia

*IMV* invasive mechanical ventilation, *NIV* non-invasive ventilation, *MI-E* mechanical insuflator-exsufflator, *GERD* gastroesophageal reflux disease

**Table 4:** Assessment and management before delivery

	**N. of patients** (94 cases)
**Pulse-Oximetry**	**89 (94,7%)**
SpO_2_ < 95% at room air	6
**Spirometry**	**86 (91,5%)**
FVC < 50% and ≥ 30% of predicted	5
FVC < 30% of predicted	6
**PCF**	**42 (44,7%)**
PCF < 270 L/min	8
**Carbon dioxide level assessment**	**35 (37,2%)**
**Diurnal** hypercapnia (PaCO_2_ ≥ 50 mmHg)	0
**Sleep respiratory studies**	**7 (7,4%)**
Altered sleep respiratory study	0
**History of weak cough or recurrent respiratory illness**	**4 (4,2%)**
**Preoperative training in NIV**	**7 (7.5%)**
**Retraining in NIV**	**8 (8,5%)**
**Preoperative training in cough assistance**	**10 (10.6%)**
**Retraining in cough assistance**	**6 (6,4%)**
**Echocardiogram**	**35 (37,2%)**
Ejection Fraction < 35%	0
**Preanesthetic location**	
Inpatient Ward	**90 (95,7%)**
High Dependency Unit	**4 (4,2%)**

*FVC* forced vital capacity, *MV* mechanical ventilation, *NIV* non-invasive ventilation, *ICU* intensive care unit

Twenty-four (25.5%) [CI 95% 15.1–33.3]) patients were at risk of developing PCs and half of them presented more than one risk factor. Pre-existing respiratory device dependence and FVC < 50% of predicted value were the most frequently reported risk factors (Table 5). Among pregnant women with pre-existing technology dependence seven used NIV and MI-E, three used only NIV, and one used only MI-E. Training or re-training in NIV or in cough assistance were required respectively in 10 and in 9 cases.

**Table 5:** Percentage of pregnant women with respiratory risk factors for pulmonary complications

**Respiratory risk factors for pulmonary complications**	**% of patients (N. of patients)** (94 cases)
**FVC < 50% of predicted value**	**11.7% (11)**
**Long-term NIV**	**10.6% (10)**
**Use of cough assistance techniques at home**	**8.5% (8)**
**PCF < 270 L/min**	**8.5% (8)**
**SpO2 < 95% at room air**	**6.4% (6)**
**Cobb angle ≥ 50°**	**5.3% (5)**
**Gilardeau dysphagia score > 1**	**5.3% (5)**
**History of weak cough or recurrent respiratory illness**	**4.2% (4)**
**Diurnal or nocturnal hypercapnia** (PaCO2 ≥ 50 mmHg)	**0**
**Central or obstructive apneas**	**0**

*FVC* forced vital capacity, *NIV* non-invasive ventilation, *PCF* peak cough flow, *SpO2* 0xygen saturation

### Details of delivery and post-delivery management

Mean gestational age at delivery was 36.2 ± 6.5 weeks. Cesarean sections were performed in 72 (76.6%) cases, including nine women who underwent urgent cesarean section (Table 6). Urgent cesarean sections were due to lack of progress in labor in five cases and to premature rupture of membranes in two cases; the cause was not reported in the other two cases. Among the 22 patients, who underwent vaginal delivery, vacuum assisted extraction was performed in five patients. Vacuum-assisted vaginal delivery was due to low uterine contractions in four cases and to head-pelvic disproportion in one case.

**Table 6:** Details of delivery, postpartum care and outcomes

	**Patients** (94 cases)
**Gestational age at delivery** (Mean ± SD)	**36,2 ± 6,5**
**Mode of delivery,** Number (%)	
Scheduled Cesarian Section	**63 (67,0%)**
Vaginal Delivery	**22 (23,4%)**
Vacuum-Assisted Vaginal Delivery	**5 (20,8%)**
Induced Labor	**10 (10,6%)**
Urgent Cesarian Section	**9 (9,6%)**
**Epidural Analgesia** (N. of patients)	**15 (15,9%)**
**Disposition following birth** (N. of patients)	
General Ward	**70 (74,5%)**
ICU	**12 (12,8%)**
HDU	**12 (12,8%)**
**Use of MV and MI-E after delivery** (N. of patients)	
NIV only	**7 (7,4%)**
NIV and MI-E	**10 (10,6%)**
Invasive Ventilation	**6 (6,4%)**
**Post-delivery analgesia** (not exclusive) (N. of patients)	
Epidural analgesia	**28 (29,8%)**
Acetaminophen or NSAIDS	**85 (90,4%)**
Opioids ev	**33 (35,1%)**
**Peripartum complications** (n. of patients)	
Surgical complications	**9 (9,6%)**
*Pulmonary complications:*	***10 (10,6%)***
**-** Atelectasis	
**-** secretion retention	*10*
**-** respiratory failure	
**-** prolonged intubation	*1*
Hypothermia	**1 (1,1%)**
PROM	**5 (20,8%)**
Myasthenic crisis	**1 (1,1%)**
**Birth Weight (**g)	**3010,6 ± 419,9**
**Apgar** at** 1** min	**8.41 ± 1.15**
**Apgar** at** 5** min	**8.99 ± 0.81**
**ICU LOS after delivery** (Days), Median (1^st^ and 3^rd^ Interquartile)	**2 [1, 2]**
**HDU LOS after delivery** (Days), Median (1^st^ and 3^rd^ Interquartile)	**1 [1–1.75]**
**HospitaL LOS after delivery** (Days), Mean ± SD	**5,54 ± 4,98**

*MV* mechanical ventilation, *MI-E* mechanical insufflator–exsufflator, *NIV* non-invasive ventilation, *NSAIDs* Nonsteroidal anti-inflammatory drugs, *PROM* premature rupture of membranes, *ICU* intensive care unit, *HDU* High Dependency Unit, *LOS* length of stay, *SD* standard deviation

After delivery 17 women required NIV 44.8 (± 30.5) hours with or without MI-E. Twenty-four patients were admitted to ICU or HDU after labor. ICU and HDU median length of stay (LOS) were respectively 2 and 1 day. Overall hospital long of stay (LOS) was 5.54 days.

### Complications and patient’s outcome

Ten patients (10.6% [CI 95% 3.9–15.4]) developed postpartum PCs (Table 6). Nine of them were at risk for PCs. As a consequence, pulmonary complication rate among the 24 patients at risk for PCs was 37.5%. These patients suffered from progressive muscular dystrophies (1 myotonic, 3 limb-girdle muscular dystrophies), spinal muscular atrophy (2 type II and 1 type III) and myasthenia gravis (1 patient); one suffered from neuromuscular disorder of unknown etiology. Seven of them presented more than one risk factor and had respiratory device dependency before delivery. All these nine women used NIV and MI-E after cesarean section. Two of them also required NIV during cesarean section under spinal anesthesia. Patient with myasthenia gravis was also treated with intravenous immunoglobulin for three consecutive days.

Among the other 70 patients not at risk for PCs only one woman presented pulmonary complications. This woman suffered from spinal muscular atrophy type 2. She did not undergo neither NIV nor MI-E after delivery.

The most frequent PCs was bronchial secretion retention, which was present in all patients. All ten patients, who presented PCs, underwent caesarean section and were admitted to ICU after the procedure. Five of them underwent general anaesthesia and were invasively ventilated at least for four hours after cesarean section. None of the analyzed patients underwent re-intubation, tracheostomy or death during hospitalization. No complications related to the use of MI-E or NIV was described in these women before and after delivery.

Patients with respiratory risk factors presented higher percentage of pulmonary complications (9 vs 1), higher ICU or HDU admission rate (19 vs 5), longer ICU LOS (2.8 ± 4.5 days vs 1.4 ± 0.5 days) and longer hospital LOS (8.5 ± 6.8 days vs 4.9 ± 1.9 days) than those without respiratory risk factors (Table S1, available in online supplementary material).

Surgical complications were also described in nine women. In particular, two women experienced dehiscence of surgical suture and one case of surgical site infection was reported. In addition, postdelivery hemorrhage was described in five women and there was one case of uterus atony after labor. Other complications were illustrated in (Table 6).

### Neonate’s outcome

The average neonatal weight at birth was 3010 g. Their average Apgar score at one minute was 8.41 and it increased to 8.99 at five minutes. No neonate was diagnosed with congenital myotonic dystrophy. Only one newborn needed endotracheal intubation, probably due to the narcotics used for mother awake fibreoptic assisted intubation.

Patients with respiratory risk factors showed lower Apgar scores at one (7.2 ± 1.4 vs 8.6 ± 0.9) and five minutes (8.5 ± 1 vs 9.1 ± 0.7), and lower neonatal weight (2765.5 ± 354.2 g vs 3077.1 ± 398.2 g) than those without respiratory risk factors.

### Details of anesthesia

The large majority of patients who underwent cesarean section. (65 out of 72) were managed with regional anesthesia. General anesthesia was only performed in seven cases and in five of them difficult intubation was recorded. In particular, three women required awake fiberoptic-assisted endotracheal intubation and two patients underwent videolaringoscopy intubation. Considering predictors of difficult intubation assessed during pre-operative evaluation, all thesewomen showed a high Mallampati’s oropharyngeal classification, respectively four women with class IV and one with class III. Moreover, two patients had intercisor gap < 4 cm and two referred an history of difficult intubation. Consequently, four women had more than one finding predictive of difficult endotracheal intubation. In case of general anesthesia, the use of halogenated agents was averted in all patients with myopathies to avoid rhabdomyolysis. In these patients total intravenous anesthesia with ultra-short acting drugs, such as propofol and remifentanil were performed. Rocuronium was administered to induce muscles paralysis, and train-of-four monitor was used to measure the degree of neuromuscular blockade Neuromuscular block had been always reversed with sugammadex to prevent postoperative residual curarization (PORC). Acetaminophen was administered as post-delivery analgesia in most patients, while 33 (35.1%) patients received i.v. morphine. Acute rhabdomyolysis or PORC were never reported in these women. Other details about anesthetic management and post-delivery analgesia are shown in Table S2, available in the online supplementary material.

## Discussion

This pilot study showed the feasibility and safety of applying a protocol for screening and preventing PCs in pregnant NMDs women. We found that, among a population of 94 of these patients, 25% women were at risk for PCs. Despite a PCs rate of 37% was observed in this subgroup, maternal and neonatal outcome were favorable, and none of them needed reintubation nor tracheostomy. To the best of our knowledge, this is the first study reporting the risk of developing PCs and the patient’s outcome in pregnant NMDs women. Furthermore, our data suggested that MI-E might be safely used in these patients in combination with NIV.

During pregnancy, intrauterine growth of the fetus impairs diaphragm function and increases respiratory workload, further worsening alveolar hypoventilation and cough impairment [4, 19]. Respiratory assessment before delivery is strongly recommended in NMDs women, because it allows to identify women who need for specific management to prevent and treat PCs [4]. Our literature review allowed us to identify only isolated case reports of pregnant women with NMDs (i.e., polio, spinal muscular atrophy, limb-girdle muscular dystrophy, amyotrophic lateral sclerosis and mitochondrial myopathies) who had severe respiratory muscle weakness before pregnancy [9, 19–24]. These studies reported that different forms of NMDs share common complications (i.e., alveolar hypoventilation and bronchial clearance impairment). They also highlighted that the use of NIV in the peri-partum period may avert PCs enabling the delivery of wholesome neonates despite the pre-conception respiratory compromise (i.e., FVC less than 10% of predicted, very low peak cough flow rates, severe kyphoscoliosis, non-invasive ventilatory support before pregnancy). The results of our study confirm and expand in a large cohort the findings of isolated case reports. Indeed, we showed that when NMDs pregnant women with severe respiratory muscle weakness were trained in the use of NIV and/or mucus clearance techniques before pregnancy, and they used NIV in the peri-partum period, mother and neonatal outcome was favourable. However, the subgroup with respiratory risk factors had a higher percentage of PCs, higher ICU or HDU admission rate, longer ICU and hospital LOS than those without respiratory risk factors.

As far as we know, this is the first study that reports the use of MI-E in pregnant women. All ten pregnant women with pulmonary complications reported secretions retention and nine of them used MI-E. No complications related to MI-E was described in our patients before and after delivery. Thus, our data suggest that MI-E might be safely used in combination with NIV to prevent and treat secretion retention during pregnancy.

Regarding delivery strategies, vaginal delivery is not contraindicated in women with NMDs, as uterus is smooth muscle autonomically innervated and it should not be affected by the majority of these disorders. However, uterine muscle abnormalities are described in myotonic dystrophy type [13, 29] and ineffective contractions are reported in some spinal muscular atrophy women [30, 31]. On the other hand, in NMDs women weakness in pelvic and abdominal muscles are frequently reported [11, 12, 32], and pelvic anatomy may be altered [32, 33]. All these abnormalities may impede normal delivery leading to assisted vaginal delivery or cesarean section [29–34]. In our cohort 77% of pregnancies underwent cesarean section and among the remaining 22 patients, who underwent vaginal delivery, vacuum assisted extraction was performed in five patients.

Concerning anesthetic strategies for cesarean section in NMD patients with decreased pulmonary function, regional anesthesia should be preferred to general anesthesia in order to reduce respiratory complication [3, 4, 14, 15]. However, severe scoliosis may be sometimes present in these women, making difficult to perform neuroaxial blockade [5–7, 11]. In our study only five patients had severe scoliosis, and among patients who required caesarean section all but seven underwent regional anesthesia, confirming that in pregnant women with NMDs regional anesthesia is the first-choice anesthesia technique [4]. In addition, epidural analgesia was administered in 15 out of 22 patients who underwent vaginal delivery.

This pilot study has several limitations. Firstly, there is no control group. However, NIV and MI-E are used as a first-line treatment in our centers for all patients with NMDs to prevent PCs. Consequently, a prospective randomized controlled trial would be difficult to carry out for ethical reasons. Secondly, a relatively low number of patients with different NMDs was included in the study. However, NMDs are rare diseases and to date this is the largest study that reported pre-operative training and postoperative use of NIV and MI–E to prevent PCs in pregnant women with NMDs. Moreover, studies concerning respiratory impairment in these patients showed that hypoventilation and bronchial secretion retention are common features in different NMDs [6, 19–24]. Thirdly, the study doesn’t comprehend statistical tests, due to low number of patients and the heterogeneity of disorders with variable disease severity. As a consequence, the endpoint of this study is only to describe the percentage of pregnant women with respiratory risk factors and the populations outcomes. Unfortunately, we did not find in the literature a valid comparator concerning the rate of PCs in pregnant women with NMD. Lastly, the positive outcomes of this study may also be due to the peculiarities of the medical centers where NMDs pregnant patients were treated, particularly to the presence of trained multidisciplinary teams and the availability of an ICU or HDU to manage a potentially high risk post-delivery course [14, 15, 35, 36].

In conclusion, only a quarter of NMDs pregnant women enrolled in our study were at risk of PCs and needed MI-E and NIV training. Despite their level of baseline complexity was quite high and an elevated PCs rate was observed in the subgroup of patient at risk for respiratory complications, maternal and neonatal outcome were globally favourable. Before recommending the adoption of this protocol more data from larger prospective multicenter studies are needed, possibly evaluating cohorts of patients with specific diseases.

### Supplementary Information

**Additional file 1. Table S1. **Baseline characteristics, clinical data and outcomes of patients with and without respiratory risk factors. **Table S2.** Anesthetic technique and airway management in case of cesarean section. **Table S3.** Consort flow diagram.

## Data Availability

The datasets used and/or analyzed during the current study are available from the corresponding author on reasonable request. Data supporting the results of this study are available from the AON SS Antonio e Biagio e Cesare Arrigo, Alessandria, Italy, EU but the availability of these data is restricted, and they are used under license from the current study and therefore not publicly available. However, data may be obtained from the authors upon reasonable request and with the permission of the Ethics Committee of the AON SS Antonio e Biagio e Cesare Arrigo, Alessandria, Italy, EU.
