# Supplementary material for: Peri-partum respiratory management of pregnant women with neuro-muscular disorders: a prospective observational study (IT-NEUMA-Pregn study)
Source: BMC Anesthesiol. 2023 Oct 13;23:342. doi: 10.1186/s12871-023-02307-6 (PMC10571352; doi:10.1186/s12871-023-02307-6)
Supplement: Supplementary file 1 — Additional file 1. Table S1. Baseline characteristics, clinical data and outcomes of patients with and without respiratory risk factors. Table S2. Anesthetic technique and airway management in case of cesarean section. Table S3. Consort flow diagram. [file 12871_2023_2307_MOESM1_ESM.docx]

**ONLINE SUPPLEMENTARY MATERIAL**

**Table S1. Baseline characteristics, clinical data and outcomes of patients with and without respiratory risk factors**

| Baseline variables | Women without respiratory risk factors  (70 patients) | Women with respiratory risk factors  (24 patients) |
| --- | --- | --- |
| Age (years) | 33.1 ± 4.8 | 31.8 ± 3.6 |
| Weight (Kg) | 73.4 ± 9.6 | 68.7 ±10.1 |
| Height (cm) | 163.8 ± 7.0 | 161.1 ± 5.4 |
| Cesarian section (N. of patients) | 51 (72,8%) | 21 (87,5%) |
| Vaginal delivery (N. of patients) | 19 (27,1%) | 3 (12,5%) |
| Elective cesarian surgery (N. of patients) | 44 (62,8%) | 19 (79,1%) |
| Emergent cesarian surgery (N. of patients) | 7 (10%) | 2 (9,5%) |
| Spinal anesthesia (N. of patients) | 42 (60%) | 12 (50%) |
| Combined spinal-epidural anesthesia (N. of patients) | 5 (7,1%) | 3 (12,5%) |
| Epidural anesthesia (N. of patients) | 2 (2,8%) | 1 (4,1%) |
| General anesthesia (N. of patients) | 2 (2,8%) | 5 (20,8%) |
| Epidural analgesia (N. of patients) | 12 (17,1%) | 3 (12,5%) |
| Morfine i.v. after surgery (N. of patients) | 23 (32,8%) | 8 (33,3%) |
| Pulmonary complications (N. of patients) | 1 (1,4%) | 9 (37,5%) |
| ICU or HDU admission rate (N. of patients) | 5 (7,1%) | 19 (79.2%) |
| ICU LOS (days) | 1.4 ± 0.5 | 2.8 ± 4.5 |
| Hospital LOS (days) | 4,9 ± 1.9 | 8.5 ± 6,8 |
| Neonatal weight (g) | 3077.1 ± 398.2 | 2765.5 ± 354.2 |
| Apgar at 1 min | 8.6 ± 0.9 | 7.2 ± 1.4 |
| Apgar at 5 min | 9.1 ± 0.7 | 8.5 ± 1.0 |

Legend: ev, endovenous; NIV, non-invasive ventilation; MI-E, mechanical insufflator–exsufflator; ICU, intensive care unit; LOS, length of stay.

**Table S2. Anesthetic technique and airway management in case of cesarean section**

|  | N. of patients (72 cases) | |
| --- | --- | --- |
| Premedication with Midazolam | **1** | |
| Regional Anesthesia | **65 (90,3%)** | |
| Spinal anesthesia | 54 | |
| Epidural anesthesia | 3 | |
| Combined Spinal-Epidural anesthesia | 8 | |
| General Anesthesia | **7 (9,7%)** | |
| Halogenated agents | 2 | |
| TIVA | 5 | |
| Drugs (not exclusive) | General anesthesia | Spinal anesthesia |
| Propofol | 7 | - |
| Midazolam | 1 | - |
| Fentanyl | 7 | 27 |
| Suphentanyl | 0 | 12 |
| Morphine | 0 | 28 |
| Rocuronium | 4 | - |
| Sugammadex | 4 | - |
| Airway Management |  | |
| Standard ETI with a conventional laryngoscope | 2 | |
| Fiberoptic assisted intubation | 3 | |
| Videolaringoscopy intubation | 2 | |

**Legend**: TIVA, total intravenous anesthesia; NIV, non-invasive ventilation; LMA, laryngeal mask airway; ETI, endotracheal intubation.

**Table S3. Consort flow diagram**

**
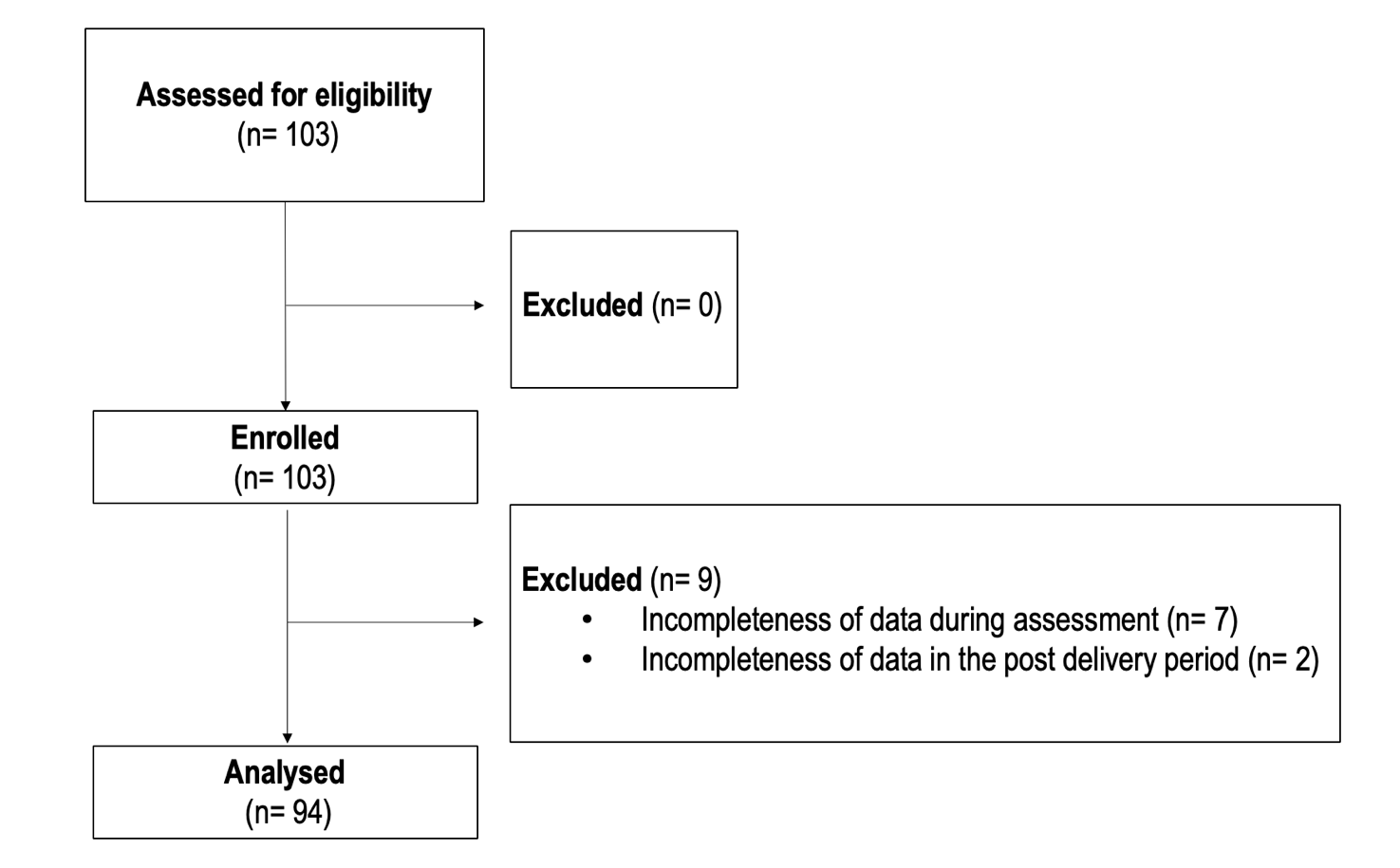
**
